# Measuring progress towards Sustainable Development Goal 3.8 on universal health coverage in Kenya

**DOI:** 10.1136/bmjgh-2018-000904

**Published:** 2018-06-27

**Authors:** Edwine Barasa, Peter Nguhiu, Di McIntyre

**Affiliations:** 1 Health Economics Research Unit, Centre for Geographic Medicine Research Coast, Nairobi, Kenya; 2 Nuffield Department of Medicine, University of Oxford, Oxford, UK; 3 Health Economics Unit, School of Public Health and Family Medicine, University of Cape Town, Rondebosch, South Africa

**Keywords:** health economics, health systems

## Abstract

**Background:**

The inclusion of universal health coverage (UHC) as a health-related Sustainable Development Goal has cemented its position as a key global health priority. We aimed to develop a summary measure of UHC for Kenya and track the country’s progress between 2003 and 2013.

**Methods:**

We developed a summary index for UHC by computing the geometrical mean of indicators for the two dimensions of UHC, service coverage (SC) and financial risk protection (FRP). The SC indicator was computed as the geometrical mean of preventive and treatment indicators, while the financial protection indicator was computed as a geometrical mean of an indicator for the incidence of catastrophic healthcare expenditure, and the impoverishing effect of healthcare payments. We analysed data from three waves of two nationally representative household surveys.

**Findings:**

The weighted summary indicator for SC increased from 27.65% (27.13%–28.14%) in 2003 to 41.73% (41.34%–42.12%) in 2013, while the summary indicator for FRP reduced from 69.82% (69.11%–70.51%) in 2003 to 63.78% (63.55%–63.82%) in 2013. Inequities were observed in both these indicators. The weighted summary measure of UHC increased from 43.94% (95% CI 43.48% to 44.38%) in 2003 to 51.55% (95% CI 51.29% to 51.82%) in 2013.

**Conclusion:**

Significant gaps exist in Kenya’s quest to achieve UHC. It is imperative that targeted health financing and other health sector reforms are made to achieve this goal. Such reforms should be focused on both, rather than on only either, of the dimensions of UHC.

**Key questions**

**What is already known?**

- Tracking country progress towards Sustainable Development Goal 3 on universal health coverage (UHC) entails measuring population coverage with healthcare services, and financial risk protection (FRP).
- Previous analysis, including in Kenya, have measured service coverage (SC) and FRP separately.

**What are the new findings?**

- This paper estimates a summary measure of UHC for Kenya that combines SC and FRP, and tracks progress over a 10-year period.
- The estimated summary measure of UHC for Kenya increased from 44% in 2003 to 52% in 2013.

**What do the new findings imply?**

- The Kenyan government should increase public financing of the health sector from the current 2.2% to at least 5% of the country’s gross domestic product, and leverage this to scale up prepayment financing while reducing reliance on out-of-pocket payments.
- Kenya needs to move away from passive purchasing, and adopt strategic purchasing practices to enhance the equity, efficiency and quality of healthcare service delivery by, among others, using a systematic process to define a benefit package that all Kenyans are entitled to, and reorienting service delivery to prioritise primary healthcare.

## Background

The inclusion of universal health coverage (UHC) as one of the health Sustainable Development Goals (SDGs) has reaffirmed its position as a key global health priority.^1^ The goal of UHC is to ensure that every citizen of a country has access to quality healthcare services that they need without the risk of financial ruin or impoverishment.^2^ Progress towards UHC entails the expansion of the range of services that are financed from pooled funds, the reduction of the proportion of total healthcare costs that are paid for by out-of-pocket (OOP) payments and the expansion of the proportion of the population that benefits from these two (service coverage (SC) and financial risk protection (FRP)).^3^

The installation of UHC as a global and country health policy goal has highlighted the need to measure it, and to track progress over time.^4–6^ The UHC SDG goal (goal 3.8) has two indicators, SC (indicator 3.8.1) and FRP (indicator 3.8.2).^7^ WHO, the World Bank (WB) and Wagstaff *et al* (2015) have further proposed the development of a summary metric of UHC that brings together these two dimensions.^4 6^ The idea is that such a metric will have utility in assessing UHC in a country, and in tracking progress over time, as well as providing an opportunity for cross-country comparison.^4 6^

Kenya has made a commitment to achieve UHC by 2022. The country’s healthcare system is pluralistic, with service provision provided by both public and private healthcare facilities in almost equal measure. The public healthcare delivery system is organised into four tiers, namely community, primary care, county referral and national referral.^8 9^ The health system is financed by revenues collected by (1) The government (national and county) through taxes and donor funding. (2) The National Hospital Insurance Fund (NHIF) through member contributions. (3) Private health insurance companies through member contributions. (4) OOP spending by citizens at points of care. Purchasing of healthcare services is carried out through: (1) Supply-side subsidies to public facilities by national and county governments; for instance the county departments of health provide line budgets to county hospitals to finance service delivery to citizens within the county. (2) The NHIF, which contracts public and private healthcare facilities in Kenya and pays them for services provided to its enrolled members. (3) Private health insurance companies that contract private healthcare facilities and pays them for service provided to their enrolled members.^10 11^ Table 1 outlines key health financing indicators for the country between 2002 and 2013.^12^

**Table 1:** Selected health financing indicators for Kenya

	2002/2003	2005/2006	2008/2009	2013/2014	2015/2016
Health insurance coverage^15 43^	9.7%	NA	10.0%	17.1%	19%
Percentage of THE financed by public sources^12 44^	29.6%	29.3%	28.8%	33.5%	37%
Percentage of THE financed by donors^12 44^	16.4%	31.0%	34.5%	24.7%	23.4%
Percentage of THE financed by private sources^12 44^	54.0%	39.3%	36.7%	40.6%	39.6%
Percentage of THE paid for through out-of-pocket expenditure^12 44^	NA	NA	25.1%	26.6%	26.1%
THE per capita (US$)^12 44^	51.2%	59.5%	66.3%	77.4%	78.6%
Government health expenditure as percentage of total government expenditure^12 44^	7.9%	5.1%	4.8%	6.1%	6.7%
THE as a percentage of GDP^12 44^	5.1%	4.7%	5.4%	6.8%	5.2%
Public expenditure on health as a percentage of GDP*	1.5%	1.4%	1.6%	2.3%	2.2%

*Source author computation from the 2013/2014 and 2015/2016 Kenya National Health accounts.

GDP, gross domestic product; THE, total health expenditure.

Several issues are apparent: (1) The Kenyan health system is heavily dependent on donor funding. (2) Private sources of financing, most of which is OOP, consistently contributes the single largest share of total health expenditure (THE). OOP spending has increased as a proportion of THE. (3) The Kenyan government has consistently underfunded and allocated a very small share of its resources to the health sector.

Since its independence in 1963, Kenya has undertaken several health financing reforms aimed at extending the proportion of the population that has FRP(see box 1). Despite these reforms, Kenya continues to fall short in attaining the UHC goals of access to quality healthcare services, and FRP. For instance, a recent assessment estimated that the level of effective coverage with key maternal and child health interventions in Kenya was 50.9%.^13^ Another assessment estimated that the incidence of catastrophic healthcare spending in Kenya was 4.52%, with 453 470 people pushed into poverty annually due to OOP spending.^14^ Further, health insurance coverage in Kenya is low (19%) and inequitable.^15 16^

Box 1 **Overview of key health financing reforms in Kenya^14 34 45 46^**

**User fee reforms**

**1963**: User fees, that had been previously introduced by the colonial government, were abolished; health sector was then predominantly tax financed.

**1989**: User fees reintroduced in public hospitals and peripheral health facilities (health centres and dispensaries) that offer outpatient primary healthcare services. User fees abolished later that year due to social justice concerns.

**1992**: User fees reintroduced again because of budgetary constraints.

**2004**: User fees abolished in public peripheral health facilities, except for a flat registration fee of Kenyan shillings (KES) 10 (US$0.1) in dispensaries and KES20 (US$0.2) in health centres. Public hospitals continued to charge user fees under a cost-sharing arrangement where hospitals received partial supply-side subsidies from the central government, and charged fees to users of healthcare services.

**2013**: After the election of a new government, user fees were completely abolished in health centres and dispensaries. A free maternity programme was introduced that removed user fees for deliveries in all public facilities.

**Health insurance reforms**

**1966**: The National Hospital Insurance Fund (NHIF) was established as a department of the Ministry of Health (MOH) to provide health insurance cover to the formal sector. Salaried individuals contributed a flat premium rate and were entitled to an inpatient benefit package.

**1972**: The law that governs the NHIF was amended to extend insurance coverage to individuals in the informal sector.

**1990**: The law was further amended to introduce a graduated premium payment structure.

**1998**: The original law was repealed and replaced with the NHIF Act of 1998 that transformed the institution from a department of the MOH to an autonomous state corporation.

**2015**: The NHIF Act was amended to revise premiums upwards. The NHIF expanded its benefit package from inpatient only, to include outpatient services as well as a raft of what has been termed special packages (radiology, cancer care, ambulance services, surgical care, chronic care, maternity care, overseas travel, renal dialysis and kidney transplant).

The installation of UHC as a global and country health policy goal has highlighted the need to measure it and to track progress over time.^4–6^ The objective of this paper is to measure UHC and examine Kenya’s progress over time. To do this, we integrate the ideas put forward in the WHO and WB framework (WHO/WB framework) for monitoring UHC,^4^ and the proposals by Wagstaff *et al* (2015) on how to operationalise this framework, to develop a summary measure for UHC for Kenya over three time points (2003, 2008 and 2014). Findings from this analysis will provide a baseline for the two UHC SDG indicators for Kenya, and find utility for policy makers in identifying areas to prioritise for improvement to accelerate progress towards the achievement of UHC in Kenya.

## Methods

### Study framework

**Figure 1:**
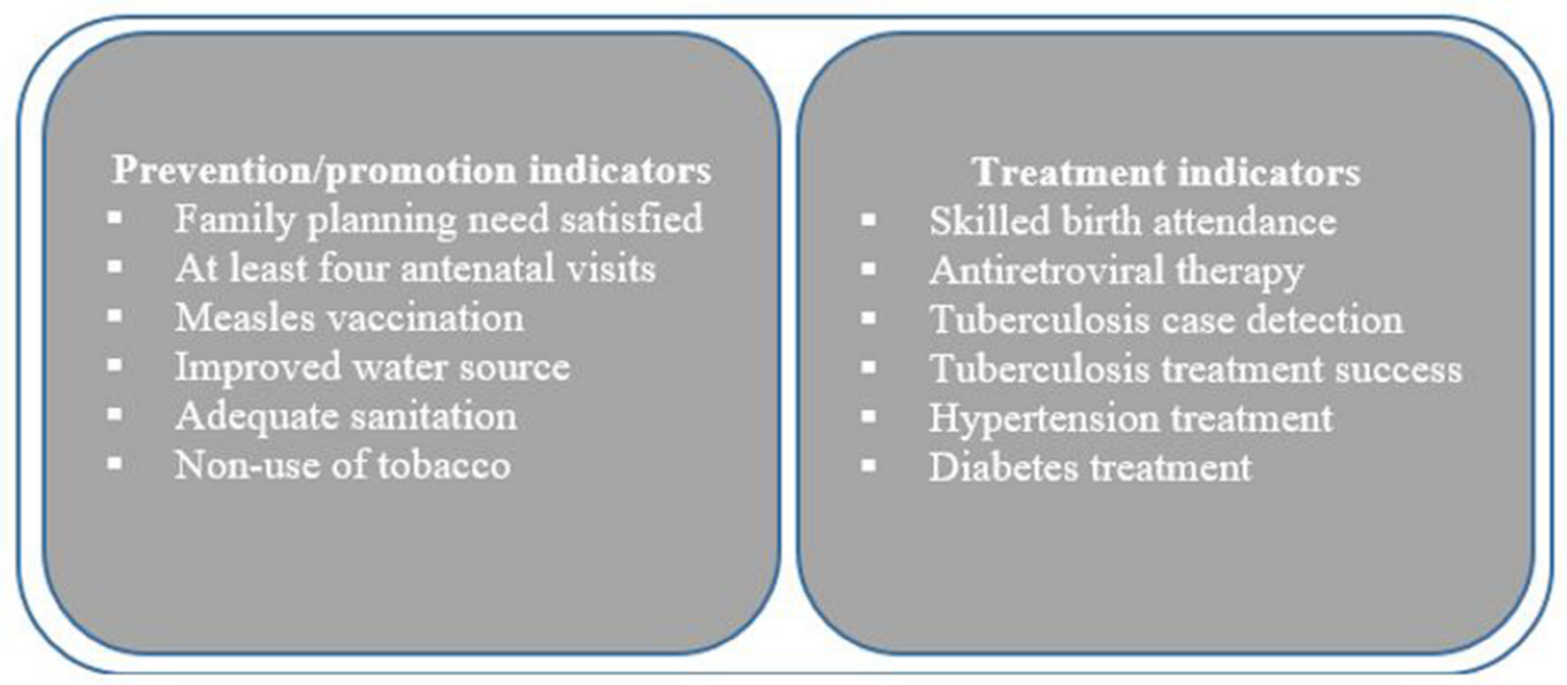
WHO and World Bank illustrative service coverage indicators.

We computed the two UHC SDG indicators that represent the two dimensions of UHC; SC and *FRP*. We further adapted the WHO/WB framework to track UHC at the country and global levels,^4^ and the proposal by Wagstaff *et al* (2015) for computationally operationalising this framework. The WHO/WB framework proposes the use of a summary measure of UHC that aggregates summary measures of two dimensions of UHC. The framework proposes that the SC summary measure should be comprised of two domains of indicators of essential health interventions—prevention/promotion and treatment.^4 5^ While the framework provides an illustrative list of SC indicators (figure 1), WHO and WB advise the use of a pragmatic and parsimonious approach in the selection of SC indicators that is guided by relevance, quality and availability of data.^4^ The framework’s FRP dimension summary measure comprises two common domain indicators—incidence of catastrophic healthcare spending and the proportion of the population that is impoverished by OOP healthcare spending.^4^ Further, because equity is at the core of UHC, the framework proposes that tracking UHC coverage should incorporate an equity analysis.

### Data

We analysed secondary data from six data sets of nationally representative household surveys in Kenya. We used three rounds of the Kenya Demographic and Health Survey (KDHS) data (2003, 2007, 2013) to analyse SC indicators, and three rounds of the Kenya Household Expenditure and Utilisation Survey (KHHEUS) (2003, 2007, 2013) to analyse FRP. KDHS is a household-based survey conducted every 5 years to collect data on marriage, fertility, family planning, reproductive health and child health. KHHEUS is a household-based survey that is conducted every 5 years to collect detailed data on sociodemographic characteristics of households and individuals, household expenditures, healthcare spending, and individual-level data on outpatient attendance (over a 4-week recall period) and inpatient hospitalisation (over a 12-month recall period). The samples for the two surveys (KDHS and KHHEUS) were drawn from a master sample developed and maintained by the Kenya National Bureau of Statistics based on a multistage sampling design. Table 2 provides the sample sizes for each survey.

**Table 2:** Descriptions of data sets

	Number of households	Number of individuals
Service coverage data sets		
KDHS 2014	36 430	31 079 (women) 12 819 (men)
KDHS 2008	9057	8444 (women) 3465 (men)
KDHS 2003	8561	8195 (women) 3578 (men)
Financial risk protection data sets		
KHHEUS 2013	33 675	1 52 566
KHHEUS 2007	8453	38 235
KHHEUS 2003	8423	38 121

Health Service Coverage.

KDHS, Kenya Demographic and Health Survey; KHHEUS, Kenya Household Expenditure and Utilisation Survey.

In line with recommendations of the WHO/WB framework, we used prevention and treatment indicators of SC.^17^ We used the following four prevention indicators: four or more antenatal visits, full immunisation, condom use and family planning. All these indicators, except for the condom use indicator, are part of the indicators recommended by the WHO/WB framework. We included the condom use indicator since HIV/AIDS is the leading cause of morbidity and mortality in Kenya, and condom use is one of the key preventive strategies that is promoted by the Kenyan government.^8^ Given that we are particularly interested in indicators that represent the efforts of the health system to promote health, we followed the approach by Wagstaff *et al* (2015) and excluded two framework indicators because they are the responsibility of agencies beyond the health sector (water and sanitation infrastructure) or involved household choices that are influenced by non-health considerations (tobacco use). We used the following health treatment indicators; whether a baby was delivered by a skilled birth attendant, whether a child with diarrhoea received the appropriate treatment and whether a child with acute respiratory infection (ARI) accessed treatment. It has been argued that these service-specific indicators do not capture the majority of treatment episodes in a health system.^6 18^ In line with the approach proposed by Wagstaff *et al* (2015), we included an indicator that captures whether a respondent to health surveys had been admitted to a hospital in the previous year. Since it is problematic to ascertain need for hospitalisation, we used the WHO Service Availability and Readiness Assessment benchmark of 10 admissions per 100 people.^6 18^ Among the SC indicators we selected, only the skilled birth attendant indicator is derived from the WHO/WB framework. We excluded the other framework indicators because data were not available in our secondary data sets. Table 3 presents the numerator and denominator definitions for each of these indicators.

**Table 3:** Numerator and denominator definition of SC interventions

	Numerator (definition of use)	Denominator (definition of need)
Prevention and promotion indicators		
Four or more antenatal visits	Number of women 15–49 years old with at least one child under 5 years, whom for their most recent birth, reported having made at least four antenatal care visits	Number of women 15–49 years old with at least one child under 5 years
Full immunisation	Number of children alive between 12 and 23 months who received the complete set of essential vaccines, that is, BCG, three doses of polio vaccine, three doses of DTP pentavalent, three doses of pneumococcal vaccine (from January 2011 onwards), and measles vaccines	Number of children alive between 12 months and 23 months
Condom use	Number of men between 15 years and 54 years and women between 15 years and 49 years who had sexual intercourse with more than one sexual partner in the past 12 months, who reported condom use in their last sexual intercourse	Number of men between 15 years and 54 years and women between 15 years and 49 years who had sexual intercourse with more than one sexual partner in the past 12 months
Family planning	Number of women 15–49 years, married or sexually active, reporting current use of any modern family planning method	Number of women 15–49 years, married or sexually active, who are fecund and reported a desire to either limit or delay pregnancy for more than 2 years
Treatment indicators		
Delivery by skilled birth attendant	Number of live births reported within 5 years of the survey, delivered by a skilled attendant (doctor, nurse or midwife)	Number of live births reported within 5 years of the survey
Appropriate treatment of diarrhoea	Number of children that had diarrhoea in the preceding 4 weeks, who were given oral rehydration therapy or increased fluids	Number of children under 5 years reported to have had diarrhoea in the preceding 4 weeks
Treatment for ARI	Number of children with symptoms of ARI that obtained treatment from a health facility or provider	Number of children under 5 years of age who had symptoms of ARI in the 2 weeks preceding the survey
Hospital admission	Number of hospital admissions by individuals in the sample in the preceding 12 months	Number of individuals in the sample

ARI, acute respiratory infection; DTP, diptheria, tetanus, pertusis.

We computed population means for each SC indicator in each of the study time points (2003, 2008, 2014). To examine inequality in coverage, we computed concentration indices for the distribution of each of the SC indicators across individuals ranked by a wealth index.^19^ To account for differences across income groups, we computed an achievement index by assigning an achievement score below the population mean to indicators that achieved high SC rates by disproportionately covering the rich, and vice versa.^6 18^ Computationally, this is achieved by multiplying the population mean by the complement of the concentration index.^6 18^

### Financial protection

We used the two common indicators of FRP. The first is the incidence of catastrophic health expenditure. We used the 40% non-food expenditure threshold popularised by WHO.^20^ We chose this threshold given that it considers the effective income remaining after basic subsistence needs have been met, rather than the total household expenditure, and therefore better approximates capacity to pay for healthcare expenditure. In line with the approach by Wagstaff *et al* (2015), we computed the complement of this proportion, in order to obtain the proportion of households that *did not* incur catastrophic healthcare expenditures and hence were deemed to have some level of FRP. We computed the population means of this indicator for each of the study time points, and the concentration index to examine inequality in FRP in each of the analysis years (2003, 2008, 2014). We then computed an achievement index to account for the distribution of catastrophic spending across wealth groups.

The second FRP indicator is the proportion of the population that is impoverished by healthcare costs. This indicator counts individuals that are pushed below the poverty line by OOP spending over a given period of time (typically a 12-month period).^21^ We used the Kenya national poverty line. However, by definition and by computation, this indicator does not count those individuals who were already living below the poverty line and hence are pushed further into poverty by OOP spending. Poor individuals need FRP from OOP spending even more than the non-poor. Given that low and middle income countries (LMICs) are characterised by high proportions of the poor individuals in the population, considering only those individuals that are pushed into poverty by OOP spending overstates FRP especially in LMICs. Therefore, in a slight departure from the impoverishment indicator as computed by Wagstaff *et al* (2015), we computed the proportion of the poor that incurred any form of OOP spending (ie, the proportion of the poor that are pushed further into poverty) and added this to the proportion of individuals (that were previously non-poor) that were pushed into poverty due to OOP spending. Our rationale is that FRP within the context of UHC must mean that poor people should not incur any form of OOP to access needed healthcare services since they are already in a vulnerable financial state. We then computed the complement of this indicator to obtain the proportion of the population that is not pushed into, or further into, poverty by OOP spending. Table 4 presents the numerator and denominator definitions for each of the FRP indicators.

**Table 4:** Numerator and denominator definition of service coverage interventions

Indicator	Numerator	Denominator
Proportion of the population that did not incur catastrophic healthcare expenditure	Number of households in the survey that did not incur OOP spending exceeding 40% of their annual non-food expenditures in the preceding 12-month period	Number of households in the survey
Proportion of individuals that were not pushed into, or further into, poverty by OOP spending	Number of non-poor individuals that where not pushed into poverty by OOP spending plus the number of poor individuals that did not incur any OOP spending	Number of individuals in the survey

OOP, out-of-pocket.

### Summary index of UHC

To compute a summary measure of UHC for Kenya, we aggregated the SC, and the FRP indicators. In line with the approach proposed by Wagstaff *et al* (2015), we computed the summary measure of UHC as follows. First, we computed the geometrical mean of prevention indicators giving each indicator equal weight as follows:

(1) $$P=\sqrt[{4}]{\left(C\times ANC\times I\times FP\right)}$$

Where *P* is the summary measure of preventive and promotive interventions, *C* is the population mean coverage with the condom use, antenatal care (ANC) is the population mean coverage with at least four ANC visits, *I* is the population mean coverage with full immunisation and family planning is the population mean coverage with family planning.

Second, in line with the approach by Wagstaff *et al* (2015), we computed the geometrical mean of the treatment indicators, giving the hospital admission indicator a weight of 50% while sharing the other 50% equally among the other treatment indicators as follows:

(2) $$T=\sqrt[{10}]{\left(A^{5}\times D^{1.67}\times ARI^{1.67}\times SD^{1.67}\right)}$$

Where *T* is the summary measure of treatment interventions, *A* is the mean population coverage with hospital admissions, *D* is the mean population coverage with appropriate treatment with diarrhoea, *ARI* is the population mean coverage with access to treatment for ARI, and *SD* is the population mean coverage with deliveries by skilled birth attendants.

Third, in line with the approach by Wagstaff *et al* (2015), we computed a summary measure for SC as the geometrical mean of the prevention and treatment indicator, giving the prevention indicator a lower weight of 25% and the treatment indicator a higher weight of 75% as follows:

(3) $$SC=\sqrt[{4}]{\left(P\times T^{3}\right)}$$

Where *SC* is the summary measure of SC, *P* and *T* are computed from equations 1 and 2, respectively. Fourth, we computed the summary measure of FRP as the geometrical mean of the two indicators, assigning equal weight as follows:

(4) $$FRP=\sqrt[{2}]{\left(CE\times IP\right)}$$

Where *FRP* is the summary measure of FRP, castrophic expenditure (*CE)* is a measure of the proportion of the population that did not incur catastrophic health spending, and *IP* is the proportion of the population that were neither pushed into poverty nor further into poverty by OOP spending.

Fifth, we computed a summary measure of UHC for each year of analysis as the geometrical mean of the SC and FRP summary measures as follows:

(5) $$UHC=\sqrt[{2}]{\left(SC\times FRP\right),}$$

**Figure 2:**
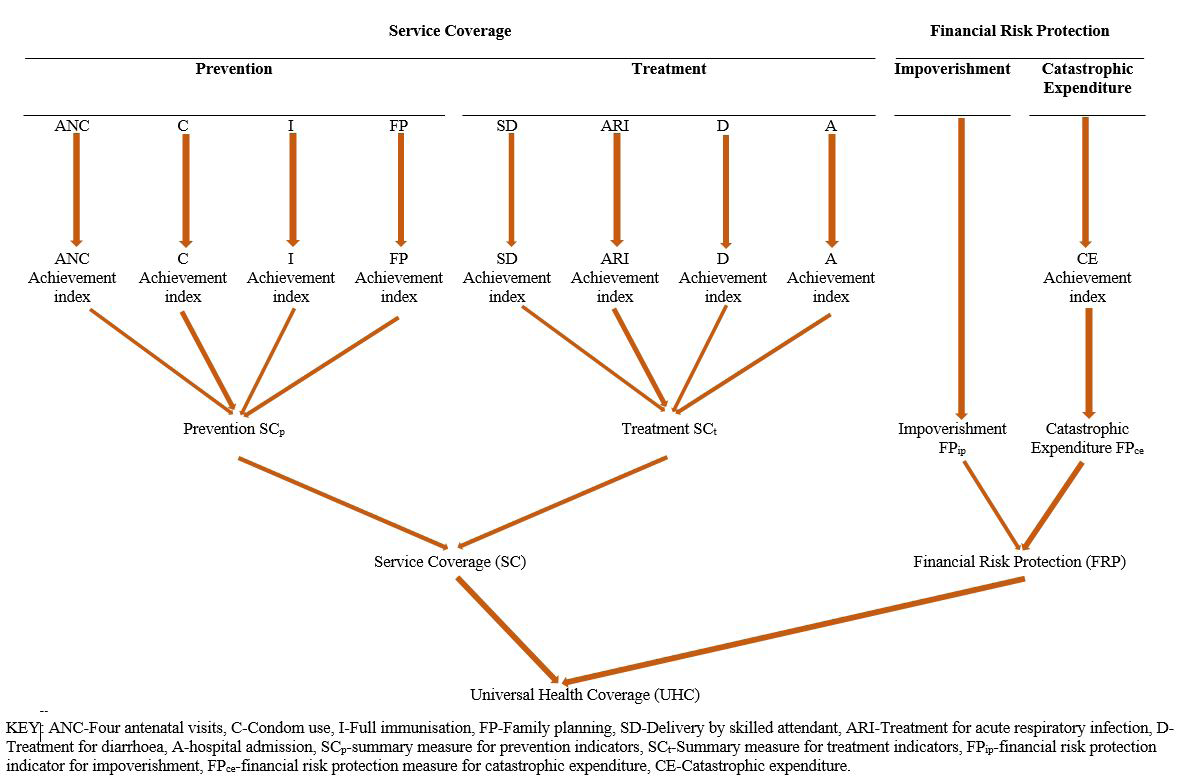
Computation of the universal health coverage (UHC) Index.

Where *UHC* is the summary measure of UHC, while *SC* and *FRP* are computed in equations 3 and 4, respectively. Figure 2 elaborates on the computation of the summary measure of UHC.

### Uncertainty and sensitivity analysis

We carried out Monte Carlo simulation with 10 000 iterations using Crystal Ball software to compute 95% CIs around the summary measures (SC, FRP, UHC). We performed sensitivity analysis on our weighting assumptions by, (1) Computing an unweighted summary measure of UHC. (2) Carrying out probabilistic sensitivity analysis on the weighting assumptions by Monte Carlo simulation with 10 000 iterations. While the base case analysis assumed that hospital admissions had a 50% weight compared with the other three curative interventions, the sensitivity analysis used a range of between no weight (lower range) and 50% weight (upper limit). Further, while the base case analysis assumed that curative interventions had 75% weight compared with preventive interventions in the computation of a summary measure of SC, the sensitivity analysis used a range of no weight (lower limit) and 75% weight (upper limit).

## Results

### Health service coverage

Table 5 outlines findings on crude population coverage with key healthcare interventions in the three analysis years. Coverage with all but one (four ANC visits) preventive care interventions generally increased between 2003 and 2014. While coverage with full immunisation in children, and family planning services increased from about 50% to over 70% over the 3 years, coverage with the other two interventions (condom use, and four ANC visits) remained very low (below 50%) over the 3 years. The distribution of coverage for all prevention interventions was inequitable (prorich) except for condom use which was propoor in 2008. Coverage for all but one (appropriate treatment for diarrhoea in children) treatment interventions increased between 2003 and 2014. The distribution of coverage with skilled delivery and hospital admissions was inequitable (prorich). The distribution of coverage with treatment for ARIs and appropriate treatment of diarrhoea in children was inequitable (prorich) in 2014, despite being propoor or equal in all the previous study years. The weighted summary index of SC, while generally low, increased from 27.65% (27.13%–28.14%)%) in 2003 to 41.73% (41.34%–42.12%) in 2014.

**Table 5:** Service coverage in Kenya

Kenya UHC indicators	2003			2008			2014		
	Mean coverage (95% CI)	CIX (95% CI)	Achievement score	Mean coverage (95% CI)	CIX (95% CI)	Achievement score	Mean coverage (95% CI)	CIX (95% CI)	Achievement score
Prevention indicators									
Condom use among men/women who had 2+ sexual partners	27% (22.4 to 31.3)	0.18 (0.09 to 0.27)	22% (18%–26%)%)	34% (28.5 to 40.3)	0.07 (−0.02 to 0.17)	32% (26.51%–37.48%)%)	43% (39.0 to 46.3)	0.05 (0.003 to 0.10)	40% (37.05%–43.99%)%)
Four or more ANC visits	36% (34.5 to 37.5)	0.04 (0.02 to 0.07)	34% (33%–36%)%)	31% (29.0 to 32.2)	0.06 (0.03 to 0.10)	29% (27.26%–30.27%)%)	36% (35.3 to 37.2)	0.06 (0.04 to 0.07)	34% (33.18%–34.97%)%)
Full immunisation in children	54% (50.1 to 57.5)	0.10 (0.06 to 0.14)	49% (45%–52%)%)	74% (69.7 to 77.3)	0.05 (0.02 to 0.08)	70% (66.22%–73.44%)%)	77% (75.7 to 79.1)	0.05 (0.04 to 0.07)	73% (71.92%–75.15%)%)
Family planning coverage	52% (48.9 to 54.1)	0.15 (0.13 to 0.17)	44% (42%–46%)%)	58% (55.6 to 60.1)	0.10 (0.08 to 0.13)	52% (50.04%–54.09%)%)	74% (72.6 to 75.3)	0.06 (0.05 to 0.07)	70% (68.24%–70.78%)%)
Prevention summary measure	40.36%		35.64% (33.53%–37.48%)%)	45.94%		42.87% (40.79%–44.82%)%)	54.52%		51.34% (49.90%–52.69%)%)
Treatment									
Appropriate treatment for diarrhoea	77% (73.0 to 80.1)	0.02 (−0.008 to 0.039)	76% (72%–78%)	89% (86.2 to 91.7)	−0.01 (−0.02 to 0.006)	90%(87.06%%–92.62%)	82% (79.9 to 83.6)	0.02 (0.004 to 0.03)	80% (78.30%–81.93%)
Access to treatment for acute respiratory infection	46% (42.3 to 48.7)	0.06 (0.03 to 0.09)	43% (40%–46%)%)	48% (43.8 to 51.6)	0 (−0.02 to 0.05)	48% (43.80%–51.60%)%))	59% (56.6 to 60.4)	0.02 (0.003 to 0.04)	57% (55.47%–59.19%)
Skilled delivery	41% (39.0 to 44.1)	0.27 (0.25 to 0.29)	30% (11%–32%)	44% (40.5 to 47.1)	0.26 (0.23 to 0.29)	32% (31.19%–34.54%)	62% (60.4 to 63.3)	0.21 (0.20 to 0.22)	49% (47.72%–50.01%)
Hospital admissions per 100 individuals	15%	0.06 (0.01 to 0.11)	14%	27%	0.12 (0.07-−0.17)	24%	28%	0.05 (0.03 to 0.07)	25%
Treatment summary measure	28.04%		25.41% (24.97%–25.84%)	39.28%		35.23% (34.49%–35.92%)	43.21%		38.95% (38.62%–39.28%)
Service coverage index (weighted)	27.65% (27.13% to 28.14%)			37.00% (36.29%% to 37.70%)			41.73% (41.34% to 42.12%)		
Service coverage index (unweighted)	30.09% (29.25% to 30.89%)			38.86% (37.86% to 39.85%)			44.72% (44.07% to 45.37%)		

ANC, antenatal care; CIX, concentration index; UHC, universal health coverage.

Table 6 outlines findings on FRP. The proportion of households incurring catastrophic healthcare spending reduced between 2003 and 2014. However, the incidence of catastrophe remained disproportionately higher among the poor in all the three analysis years. The proportion of individuals that are either pushed into, or further into, poverty increased between 2003 and 2014. While the proportion of non-poor that are pushed into poverty did not increase between 2003 and 2014, the proportion of the poor that were pushed further into poverty increased over this period. The summary measure of FRP reduced from 69.82% (69.11%–70.51%)%) in 2003 to 63.78% (63.55%–63.82%) in 2014.

**Table 6:** Financial risk protection (FRP) in Kenya

	2003		2007		2013	
	Mean (95% CI)	CIX (95% CI)	Mean (95% CI)	CIX (95% CI)	Mean (95% CI)	CIX (95% CI)
Incidence of CE (a)	16.3% (14.6% to 17.9%)	−0.13 (–0.16 to 0.09)	10.7% (9.6% to 11.9%)	−0.10 (–0.07 to 0.04)	4.52% (4.10 to 4.93%)	−0.30 (–0.35 to 0.26)
Proportion of the population pushed into poverty (b)	1.88%		1.20%		1.02%	
Proportion of the population pushed further into poverty (c)	31.17%		31.05%		38.12%	
Impoverishment indicator (b+c)	33.05%		32.25%		39.14%	
		Achievement index		Achievement index		Achievement index
FRP indicator 1: (1−a)	83.70%	72.82% (71.42% to 74.30%)	89.30%	80.37% (79.29% to 81.36%)	95.48%	66.84% (66.55% to 67.13%)
FRP indicator 2: 1−(b+c)	66.95%	66.95%	67.75%	67.75%	60.86%	60.86%
Summary measure of FRP	69.82% (69.11% to 70.51%)		73.79 (73.34% to 74.25)		63.78% (63.55% to 63.82%)	

CE, castrophic expenditure; CIX, concentration index.

### UHC Index

**Figure 3:**
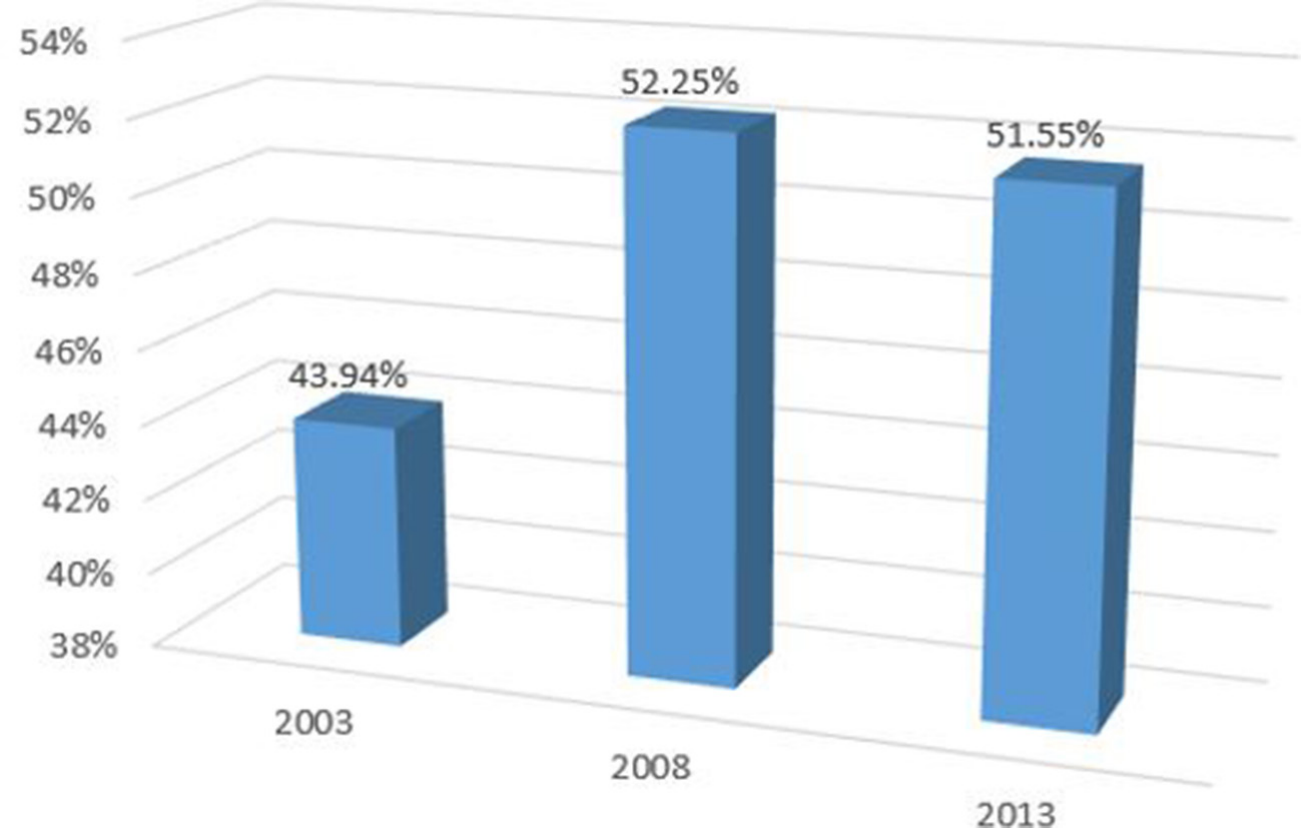
Weighted Index of universal health coverage (UHC) in Kenya.

Figure 3 outlines the weighted UHC Index for Kenya for three time periods (2003, 2007, 2017). Our analysis shows that the weighted UHC Index for Kenya was 43.94% (95% CI 43.48% to 44.38%) in 2003, 52.52% (95% CI 51.74% to 52.78%) in 2007 and 51.55% (95% CI 51.29% to 51.82%) in 2017.

### Sensitivity analysis

**Figure 4:**
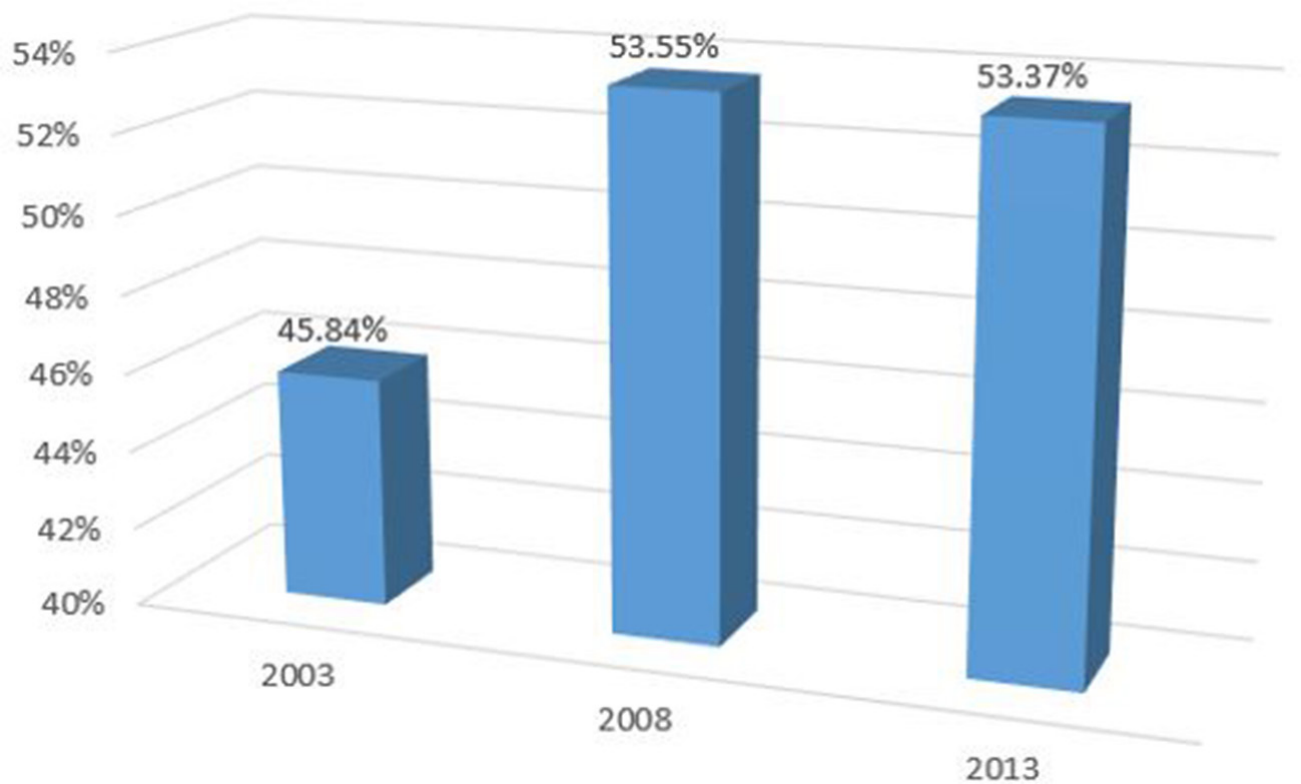
Unweighted index of universal health coverage (UHC) in Kenya.

When the UHC Index is unweighted, the index value for 2003 is 45.84% (95% CI 45.17 to 46.78), 53.55% (95% CI 52.85% to 53.80%) in 2008 and 53.37% (95% CI 52.97% to 53.80%) in 2013 (figure 4).

**Figure 5:**
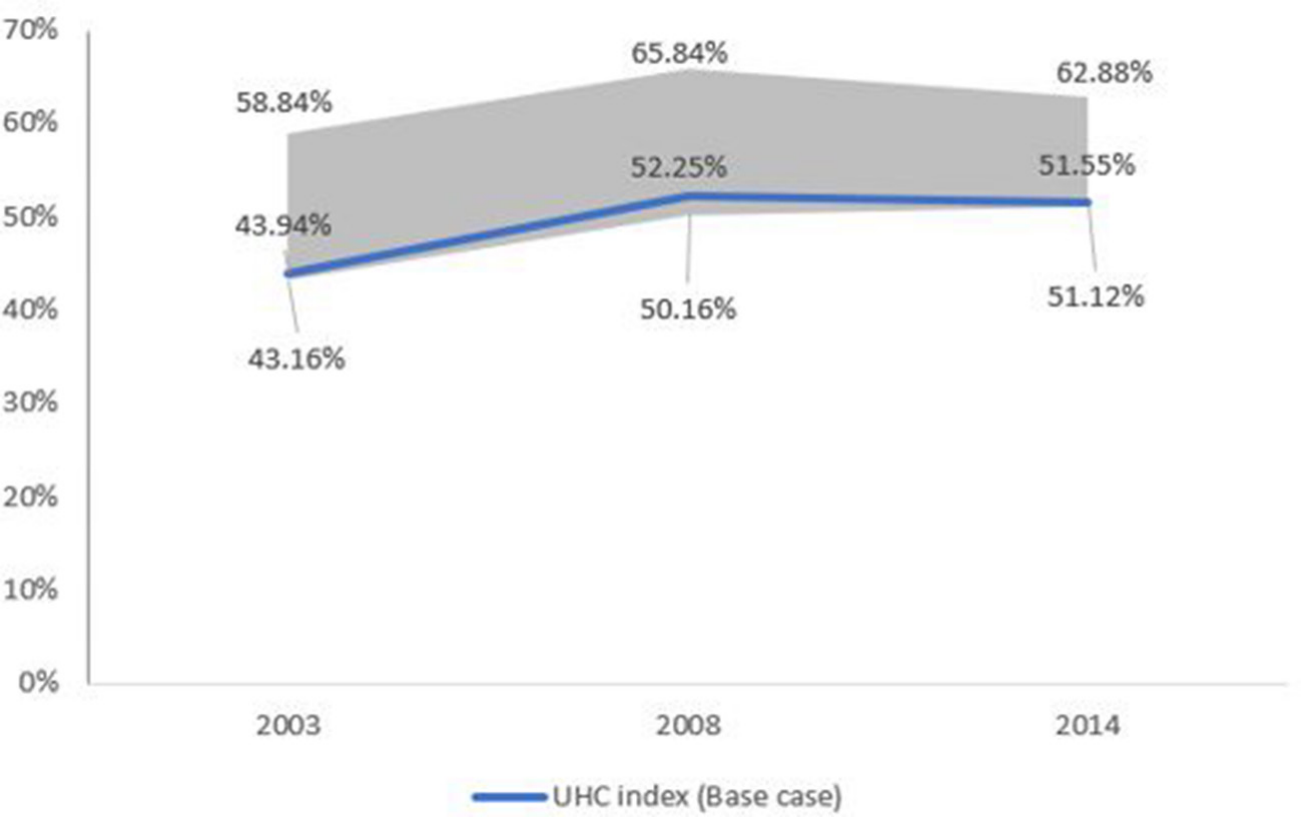
Sensitivity analysis of universal health coverage (UHC) Index.

Probabilistic sensitivity analysis of the summary UHC measure to the weighting assumptions resulted in a range of 43.16% to 58.84% in 2003, 50.16% to 65.84% in 2008 and 51.12% to 62.88% in 2013 (figure 5).

## Discussion

Given that UHC is a key global health priority, tracking country progress is crucial. In this paper, we have presented an analysis of Kenya’s progress towards UHC using an adaptation of the UHC monitoring WHO/WB framework as well as the recommendations by Wagstaff *et al* (2015) on how to operationalise this framework. Regarding SC, we make several observations. First, coverage for both preventive and curative healthcare services increased over the study years, except for the attendance of at least four ANC visits by pregnant women. It is not clear why coverage for four ANC visits did not increase. Possible reasons could include society perceptions about the importance of attending all four ANC visits. Further, even though user fees are not charged for ANC visits in public facilities, transport costs associated with facility visits could present a barrier.^14^ The increase in coverage of most indicators is perhaps indicative of the positive effect of the Kenyan government’s effort to expand access to priority healthcare services. Successive Kenyan health sector strategic plans have identified and prioritised the expansion of access to priority healthcare services to the population.^9 22^ The dramatic increase in skilled birth attendance from 44% in 2008 to 62% in 2014 illustrates the impact of government policies, in this case the removal of user fees for deliveries in all public facilities in 2013. However, despite the increase in SC, the level of coverage remains generally low. This implies that the progress is insufficient and that more needs to be done to achieve SDG goal 3.8.1 on SC. The low SC resonates with findings from Hogan *et al* that the SC index for LMICs in sub-Saharan Africa (42%) and southern Asia (53) was low.^17^ Second, while inequalities in SC reduced over the study period, significant disparities between the rich and the poor still exist. This mirrors findings reported in the 2017 WHO/WB UHC global monitoring report. For instance, the report found that only 17% of households in the poorest wealth quintile in LMICs received at least six basic health interventions versus 74% in the richest quintile.^23^ This underlines the observation that, in the absence of conscious and proactive efforts to ensure equity, policy reforms aimed at achieving UHC may preferentially benefit the well-off while excluding the poor, resulting in inequitable health systems.^24^ UHC is the ultimate expression of equity in healthcare.^3^ When UHC cannot be achieved instantly, it is crucial that progress is made equitably.^3 25^

Regarding FRP, we make the following observations. First, while there was progress on SDG goal 3.8.2 (the incidence of catastrophic healthcare expenditure reduced over the study years), the poor continued to bear a disproportionate burden of catastrophic healthcare costs. This is consistent with observations that OOP is typically regressive, and underlines the need to scale up progressive prepayment mechanisms.^26–29^ Second, while the incidence of catastrophic healthcare expenditure reduced over the study period, the proportion of Kenyans pushed into, or further into, poverty increased over the same period. The latter is observed because the proportion of poor individuals that accessed healthcare services and paid for them using OOP expenses increased over the study period. Increasing physical access to services, without improvements in coverage with prepayment health financing mechanisms has the unwanted effect of increasing population exposure to financial risk. An analysis of data from 122 countries reported that an estimated 97 individuals are pushed into poverty annually because of OOP.^30^ This underscores the need for countries to prioritise both dimensions of UHC: SC and FRP.

Regarding Kenya’s overall progress towards UHC, the computed UHC index is low and implies that the country has a long way to go to achieve UHC. While the slow progress towards UHC is perhaps symptomatic of weaknesses in all health system functions, we focus here on the health financing function. First, the Kenyan government has consistently underfunded the health sector. Government expenditure on health as a percentage of total government expenditure reduced from 8% in 2002 to 6% in 2013, while public expenditure on health as a percentage of gross domestic product (GDP) stood at only 2.3% in 2013 (table 1). While there is no magic threshold for the ideal level of public spending in healthcare, there is consensus that increased public spending in healthcare will be required for countries to achieve UHC.^31^ It has been shown that significant improvements in health outcomes and FRP become evident when the level of public spending on healthcare reaches and exceeds 5% of a country’s GDP.^31^ Second, Kenya’s health system is heavily reliant on donor funds (25.6%) and OOP payments (27%) (table 1). While donor funds increase the overall healthcare resource envelope, most of it is used to finance vertical priority programmes, specifically vaccines, malaria, HIV/AIDS and tuberculosis (TB).^12^ Given that donor funds for vertical programmes are not fungible (cannot be reallocated to other priority areas), they distort the healthcare priorities and likely introduce inefficiencies in the health system.^32^ Further, evidence from other settings has shown that donor funds crowd out domestic resources.^33^ Over-reliance on OOP exposes households to catastrophic payments and impoverishment and limits progress towards UHC. Third, Kenya’s prioritisation of the NHIF as its vehicle for attaining UHC is likely contributing to the country’s slow UHC progress. Established in 1966, the NHIF has only managed to enrol about 15% of the Kenyan population in over 50 years of its existence.^16^ Among other reasons, this is because the NHIF operates a contributory scheme where enrolment is mandatory for formal sector individuals, and voluntary for informal sector individuals.^34^ Like other LMICs, Kenya has a high poverty level (45.6%)^15^ and most of the population works in the informal sector (83.7%).^35^ International evidence shows that it is problematic to achieve high levels of coverage with a voluntary, contributory mechanism in a context of high poverty and informality levels.^36 37^ Further, health insurance coverage in Kenya is highly inequitable, where individuals in the richest quintile are more than 20 times more likely to have health insurance cover compared with those in the poorest quintile.^16^ Fourth, the overall structure of health financing contributions in Kenya has been shown to be regressive (ie, the poor contribute disproportionately more as share of their income compared with the rich).^26^ This overall regressivity of the Kenyan health financing system is contributed to by regressive OOP, NHIF and value added tax (VAT) payments.^26^ Fifth, healthcare purchasing in Kenya has been shown not to be strategic and hence compromises equity, quality and efficiency.^10 11 38^

Regarding methods for measuring and tracking UHC, we make several observations. First, regarding measuring SC, there is a need for a systematic process for selecting indicators, and a basis for assigning relative importance to selected indicators (across categories—preventive versus treatment, and within these categories). Our assumptions about indicator selection and weights in this analysis are based on similar analyses from other settings but may not reflect Kenyan decision makers’ preferences. We have, however, also presented an unweighted analysis. Second, while it is ideal to consider effective rather than crude SC, we share the experience of analysis in other settings that often data on quality are lacking in secondary data sets.^6 18 39^ To track UHC, countries must invest in collecting accurate data on SC, and on the quality of these services. Third, while typical assessments of the impoverishing effects of OOP payments only count non-poor individuals pushed into poverty, our analysis counts both non-poor and poor individuals pushed into, and further into, poverty. This is a critical omission in previous analyses because it overstates FRP, especially in LMICs, where significant proportions of the population live below the poverty line. For example, focusing only on those pushed into poverty creates the impression that 99% of the Kenyan population had FRP in 2013, while including both those pushed into, as well as those pushed further into, poverty indicates that only 61% had such protection. We have made the normative assumption that FRP, as a UHC goal, necessarily means that those without the ability to pay, specifically those that live below the poverty line (however defined—international or national), ought not to incur any form of OOP to access needed healthcare services. Any level of OOP for the poor pushes them further into poverty. A limitation of our analysis is that we did not include coverage of interventions for non-communicable diseases, and other key diseases such as HIV/AIDS and TB. This was because the data were not available in the data sets we used, and for the years of analysis. Where they were available, they were not of good quality.

Drawing from our findings, we make several recommendations that could accelerate Kenya’s progress towards UHC. First, the Kenyan government should increase public financing of the health sector. Specifically, the level of public funding for healthcare in Kenya should double, if the threshold (5% of GDP) recommended by McIntyre *et al* (2017) it to be reached. To facilitate this, a formal analysis of the fiscal space for health in Kenya is required to identify the most feasible strategies to improve public financing for healthcare in Kenya. Second, an increase in public spending should be leveraged to scale up prepayment financing while reducing reliance on OOP payments. While the government policy to abolish user fees in public primary healthcare facilities appears to have reduced direct payments at this level, accessing care in public hospitals, as well as private healthcare facilities still requires OOP payments. Providing FRP to the population accessing care using these facilities is crucial. In doing this, the country needs to reorient its health financing strategy away from a focus on contributory, voluntary health insurance, and instead recognise that increased tax funding is critical. Rather than playing both a revenue collection (by collecting premiums from individuals), and purchasing role, the NHIF’s mandate could be restricted to purchasing, with revenues collected through direct and indirect taxes by the country’s tax collecting agency, and allocated to the NHIF to purchase services for Kenyans. Third, Kenya needs to move away from passive purchasing, and adopt strategic purchasing practices to enhance the equity, efficiency and quality of healthcare service delivery. Several analyses have demonstrated that purchasing practices by the two main purchasers of healthcare services in Kenya, the NHIF and the county departments of health, are not strategic.^11 40^ Purchasing is passive when purchasers transfer funds to providers based on a predetermined budget or simply paying bills when presented, without consideration for maximising health system goals.^41^ Purchasing is strategic when decisions about what interventions to purchase (benefit package), what providers to purchase from and mechanisms for purchasing from these providers (such as provider payment mechanisms and contract arrangements), are structured to optimise health system goals of equity, efficiency and quality.^41^ While several recommendations have been offered with regard to improving purchasing in Kenya,^10 11 38^ we highlight here the need for Kenya to institute a systematic process for the development and regular updating of a harmonised benefit package that all Kenyans are entitled to. Such a benefit package should be evidence based, based on the needs of the Kenyan society, and developed using a procedure that is deemed legitimate and fair. This will ensure that healthcare resources are spent on the ‘right’ healthcare priorities and that the population has increased access to services that they need. In addition, the country should prioritise and invest in the delivery of services though primary healthcare facilities and community health systems, since there is overwhelming evidence that this is the most accessible and equitable delivery route for healthcare services.^28 42^

## Conclusion

While FRP and SC have been previously assessed in Kenya, this is the first attempt to combine these two indicators into a summary measure of UHC for the country. Our findings show that while there is progress, SC and FRP is still low and inequality remains a concern. Given that the country has committed to achieving UHC by 2030, it is imperative that targeted health financing and other health sector reforms are made to achieve this goal. Such reforms should be focused on both, rather than only either of, the dimensions of UHC.

## References

[1] United Nations. Sustainable develoment goals: 17 goals to transform our world. 2015 http://www.un.org/sustainabledevelopment/health/ (accessed 25 Mar 2016).

[2] World Health Organization. Health systems financing: the path to universal coverage. Geneva: World Health Organization, 2012 http://www.who.int/whr/2010/en/ (accessed 16 Jun 2014). 10.2471/BLT.10.078741 PMC2878164 20539847

[3] Chan M Making fair choices on the path to universal health coverage. Health Systems & Reform 2016;2:5–7. 10.1080/23288604.2015.1111288 31514652

[4] Boerma T, Evans D, Eozenou P, et al Monitoring progress towards universal health coverage at country and global levels-framework, measures and targets. Geneva Switzerland, 2014. 10.1371/journal.pmed.1001731 PMC4171369 25243899

[5] Boerma T, AbouZahr C, Evans D, et al Monitoring intervention coverage in the context of universal health coverage. PLoS Med 2014;11:e1001728 10.1371/journal.pmed.1001728 25243586 PMC4171108

[6] Wagstaff A, Cotlear D, Eozenou PH-V, et al Measuring progress towards universal health coverage. Washington, D.C, 2015.

[7] United Nations. Resolution Adopted by the General Assembly on 6 July 2017. Geneva: United Nations, 2017.

[8] Ministry of Health. Kenya Health Policy 2014-2030. Nairobi: Ministry of Health, 2011.

[9] Ministry of Health. Kenya Health Sector Strategic and Investment Plan (KHSSP) July 2013-June 2017. 2013.

[10] Munge K, Mulupi S, Barasa EW, et al A critical analysis of purchasing arrangements in Kenya: the case of the national hospital insurance fund. Int J Health Policy Manag 2017;7:1–11. 10.15171/ijhpm.2017.81 29524953 PMC5890069

[11] Mbau R, Barasa EW, Munge K, et al A critical analysis of healthcare purchasing arrangements in Kenya: a case study of the county departments of health. 2018;1. 10.1002/hpm.2604 PMC6492197 30074642

[12] Ministry of Health. Kenya National Health Accounts 2012/2013. Nairobi: Ministry of Health, 2015.

[13] Nguhiu P, Barasa E, Chuma J Determining the effective coverage of maternal and child health services in Kenya, using demographic and health survey datasets: tracking progress towards universal health coverage. Trop Med Int Heal 2017. 10.1111/tmi.12841 PMC5396138 28094465

[14] Barasa EW, Maina T, Ravishankar N Assessing the impoverishing effects and determinants of catastrophic health care payments in Kenya. Int J Equity Health 2017;16:1–14. 28166779 10.1186/s12939-017-0526-x PMC5294805

[15] Kenya National Bureau of statistics. Kenya integrated household budget survey. Nairobi: Kenya National Bureau of statistics, 2018.

[16] Kazungu JS, Barasa EW Examining levels, distribution and correlates of health insurance coverage in Kenya. Trop Med Int Health 2017;22:1175–85. 10.1111/tmi.12912 28627085 PMC5599961

[17] Hogan DR, Stevens GA, Hosseinpoor AR, et al Monitoring universal health coverage within the Sustainable Development Goals: development and baseline data for an index of essential health services. Lancet Glob Health 2018;6 10.1016/S2214-109X(17)30472-2 29248365

[18] Wagstaff A, Dmytraczenko T, Almeida G, et al Assessing latin America’s progress toward achieving universal health coverage. Health Aff 2015;34:1704–12. 10.1377/hlthaff.2014.1453 26438747

[19] Erreygers G Correcting the concentration Index. J Health Econ 2009;28:504–15. 10.1016/j.jhealeco.2008.02.003 18367273

[20] Xu K, Evans DB, Kawabata K, et al Household catastrophic health expenditure: a multicountry analysis. Lancet 2003;362:111–7. 10.1016/S0140-6736(03)13861-5 12867110

[21] O’Donnell O, van Doorslaer E, Wagstaff A, et al Analyzing health equity using household survey data - a guide to techniques and their implementation. Washington DC: The World Bank, 2008.

[22] Ministry of Health. The Second National Heath Sector Strategic Plan of Kenya (NHSSP II 2005-2010): reversing the trends. Nairobi, Kenya: Ministry of Health, 2005.

[23] World Health Organization, World Bank. Tracking universal health coverage: 2017 global monitoring report. 2017 Licence: CC BY-NC-SA 3.0 IGO.

[24] Gwatkin DR, Ergo A Universal health coverage: friend or foe of health equity? Lancet 2011;377:2160–1. 10.1016/S0140-6736(10)62058-2 21084113

[25] Norheim OF Ethical perspective: five unacceptable trade-offs on the path to universal health coverage. Int J Health Policy Manag;4:711–4. 10.15171/ijhpm.2015.184 PMC4629695 26673330

[26] Munge K, Briggs AH The progressivity of health-care financing in Kenya. Health Policy Plan 2014 29 10.1093/heapol/czt073 24107660

[27] Kwesiga B, Ataguba JE, Abewe C, et al Who pays for and who benefits from health care services in Uganda? BMC Health Serv Res 2015;15:44 10.1186/s12913-015-0683-9 25638215 PMC4324659

[28] Mills A, Ataguba JE, Akazili J, et al Equity in financing and use of health care in Ghana, South Africa, and Tanzania: implications for paths to universal coverage. Lancet 2012;380:126–33. 10.1016/S0140-6736(12)60357-2 22591542

[29] Wagstaff A, Flores G, Hsu J, et al Progress on catastrophic health spending in 133 countries: a retrospective observational study. Lancet Glob Health 2018;6:1–11. 10.1016/S2214-109X(17)30429-1 29248367

[30] Wagstaff A, Flores G, Smitz MF, et al Progress on impoverishing health spending in 122 countries: a retrospective observational study. Lancet Glob Health 2018;6:5–10. 10.1016/S2214-109X(17)30486-2 29248366

[31] Mcintyre D, Meheus F, Røttingen JA What level of domestic government health expenditure should we aspire to for universal health coverage? Health Econ Policy Law 2017;12:125–37. 10.1017/S1744133116000414 28332456

[32] Strasser R, Kam SM, Regalado SM Rural health care access and policy in developing countries. Annu Rev Public Health 2016;37:395–412. 10.1146/annurev-publhealth-032315-021507 26735432

[33] Dieleman JL, Hanlon M Measuring the displacement and replacement of government health expenditure. Health Econ 2014;23:129–40. 10.1002/hec.3016 24327240 PMC4229065

[34] Barasa EW, Mwaura N, Rogo K, et al Extending voluntary health insurance to the informal sector: experiences and expectations of the informal sector in Kenya. Wellcome Open Res 2017;2:94 10.12688/wellcomeopenres.12656.1 29387800 PMC5698913

[35] World Bank Group. Kazi Ni Kazi - Informal is not normal. Nairobi, Kenya: World Bank Group, 2016.

[36] Lagomarsino G, Garabrant A, Adyas A, et al Moving towards universal health coverage: health insurance reforms in nine developing countries in Africa and Asia. Lancet 2012;380:933–43. 10.1016/S0140-6736(12)61147-7 22959390

[37] McIntyre D, Ranson MK, Aulakh BK, et al Promoting universal financial protection: evidence from seven low- and middle-income countries on factors facilitating or hindering progress. Health Res Policy Syst 2013;11:36 10.1186/1478-4505-11-36 24228762 PMC3848816

[38] Munge K, Mulupi S A critical analysis of the purchasing arrangements in Kenya: the case of the national hospital insurance fund, private and community-based health insurance. Nairobi, 2015. 10.15171/ijhpm.2017.81 PMC5890069 29524953

[39] Mtei G, Makawia S, Masanja H Monitoring and evaluating progress towards Universal Health Coverage in Tanzania. PLoS Med 2014;11:e1001698–10. 10.1371/journal.pmed.1001698 25244395 PMC4171093

[40] Munge K, Mulupi S, Barasa EW, et al A critical analysis of purchasing arrangements in Kenya: the case of the national hospital insurance fund. Int J Health Policy Manag 2017;7:244–54. 10.15171/ijhpm.2017.81 29524953 PMC5890069

[41] RESYST. What is strategic purchasing for health? London, 2014.

[42] World Health Organization. What are the advantages and disadvantages of restructuring a health care system to be more focused on primary care services? Geneva: World Health Organization, 2004.

[43] Ministry of Health. 2013 Kenya household expenditure and utilization survey. Nairobi: Ministry of Health, 2014.

[44] Ministry of Health. Kenya National Health Accounts 2015/2016. Nairobi: Ministry of Health, 2017.

[45] Chuma J, Musimbi J, Okungu V, et al Reducing user fees for primary health care in Kenya: policy on paper or policy in practice? Int J Equity Health 2009;8:15 10.1186/1475-9276-8-15 19422726 PMC2683851

[46] Abuya T, Maina T, Chuma J Historical account of the national health insurance formulation in Kenya: experiences from the past decade. BMC Health Serv Res 2015;15:1–11. 10.1186/s12913-015-0692-8 25884159 PMC4332452

