# Correction: *Measuring progress towards Sustainable Development Goal 3.8 on universal health coverage in Kenya*

**DOI:** 10.1136/bmjgh-2018-000904corr1

**Published:** 2018-07-06

Barasa E, Nguhiu P, McIntyre D. Measuring progress towards Sustainable Development Goal 3.8 on universal health coverage in Kenya. *BMJ Glob Health* 2018;3:e000904.

In table 1, row ‘THE per capita (US$)’, the values are mistakenly expressed as percentages; they should actually be expressed as absolute numbers in USD (without the % sign).

